# Circ-SMARCA5 suppresses progression of multiple myeloma by targeting miR-767-5p

**DOI:** 10.1186/s12885-019-6088-0

**Published:** 2019-10-10

**Authors:** Haiyan Liu, Yan Wu, Shunye Wang, Jie Jiang, Chenlu Zhang, Yijing Jiang, Xingfeng Wang, Lewen Hong, Hongming Huang

**Affiliations:** Department of Hematology, Affiliated Hospital of Nantong University, 20 Xisi Road, Nantong, 226001 China

**Keywords:** Circ-SMARCA5, MM, Prognosis, miR-767-5p, Proliferation, Apoptosis

## Abstract

**Background:**

We aimed to investigate the correlation of Circ-SMARCA5 with disease severity and prognosis in multiple myeloma (MM), and its underlying mechanisms in regulating cell proliferation and apoptosis.

**Methods:**

Bone marrow samples from 105 MM patients and 36 healthy controls were collected for Circ-SMARCA5 expression measurement. And the correlation of Circ-SMARCA5 expression with patients’ characteristics and survival was determined. In vitro, the effect of Circ-SMARCA5 on MM cell proliferation and apoptosis was evaluated by altering Circ-SMARCA5 expression through transfection. Rescue experiments and luciferase assay were further performed to explore the mechanism of Circ-SMARCA5 as well as its potential target miR-767-5p in regulating MM cell activity.

**Results:**

Circ-AMARCA5 was downregulated in MM and presented a good value in distinguishing MM patients from controls and it was also negatively correlated with Beta-2-microglobulin (β2-MG) level and International Staging System (ISS) stage. Additionally, Circ-SMARCA5 high expression was associated with higher CR as well as better PFS and OS. As for in vitro experiments, Circ-SMARCA5 expression was lower in MM cell lines compared with normal cells, and Circ-SMARCA5 overexpression inhibited cell proliferation but promoted cell apoptosis in RPMI8226 cells. Rescue experiments disclosed that the effect of Circ-SMARCA5 on cell activity was attenuated by miR-767-5p, and luciferase reporter assay revealed direct binding between Circ-SMARCA5 and miR-767-5p.

**Conclusions:**

Circ-SMARCA5 is downregulated and correlated with lower β2-MG level and ISS stage as well as better prognosis in MM patients, and it inhibits proliferation but promotes apoptosis of MM cells via directly sponging miR-767-5p.

**Electronic supplementary material:**

The online version of this article (10.1186/s12885-019-6088-0) contains supplementary material, which is available to authorized users.

## Background

Multiple myeloma (MM), also called plasma cell myeloma, is a hematological malignancy characterized by proliferation of malignant monoclonal plasma cells, and its clinical manifestations include multiple bone destruction, pathological fracture, hypercalcemia, anemia, renal dysfunction and so on [1]. MM is the second most common hematological malignancy that ranks after non-Hodgkin’s lymphoma and is age-related, suggesting that with the population aging, the incidence of MM will greatly increase in the future [2]. Similar to other hematological malignancies, the common therapeutic methods for MM include chemotherapy, targeted therapy, immunotherapy and hematopoietic stem cell transplantation, while the treatment efficacy is subject to relapse of disease and refractoriness to treatments, leading to poor survival in most of the MM patients [3]. Therefore, exploring more potential targets is necessary for development of new therapies and improving patients’ prognosis in MM.

Non-coding RNAs, a category of RNA molecules not translated into proteins, are widely abundant in human genome and engage in numerous cellular processes including transcription, post-transcriptional modification and signal transduction [4]. Circular RNAs (CircRNAs) comprise a class of non-coding RNAs whose 3′ and 5′ ends are joined together to form a covalently closed loop [5]. Benefiting from the structure, circRNAs are resistant to exonuclease-mediated degradation and are more stable compared with linear RNAs [6]. Based on evidence from the past decades, circRNAs play critical roles in gene regulation by sponging micro RNAs (miRNAs) and regulating protein translation, thereby involve in pathogenesis of various diseases, and their expression patterns and mechanisms in some solid tumors have been revealed [7]. As for hematological malignancies, circRNAs participate in development and progression of diseases and serve as valuable biomarkers for diagnosis and prognosis in patients, whereas little is known with respect to circRNAs in MM [8–10].

CircRNA SWI/SNF-related matrix-associated actin-dependent regulator of chromatin subfamily A member 5 (Circ-SMARCA5) is encoded by SMARCA5 gene that is located on chromosome 4 (location: NC_000004.12 (143,513,463..143557489)) [11]. Circ-SMARCA5 is previously shown to participate in carcinogenesis by inhibiting cell proliferation, migration and invasion in several solid tumors including hepatocellular carcinoma and cervical cancer [12–14]. Whereas in hematological malignancies, the role of Circ-SMARCA5 is unknown. One previous study reports that Circ-SMARCA5 is involved in the regulation of cell cycle and alterations of chromatin structures, and dysregulation of SMARCA5 gene is presented in CD34+ hematopoietic progenitors of hematological malignancy, and we speculated that Circ-SMARCA5 might participate in etiology and work as a potential biomarker of MM, a typical hematological malignancy [11, 15]. Therefore, the purpose of this study was to investigate the correlation of Circ-SMARCA5 with disease severity and prognosis in MM patients as well as its underlying mechanisms in regulating cell proliferation and apoptosis in MM cells.

## Method

### Participants

One hundred and five de novo MM patients treated at the Affiliated Hospital of Nantong University between Jan 2015 and Dec 2016 were consecutively enrolled in this study. The inclusion criteria were: (1) newly diagnosed as MM according to 2014 International Myeloma Working Group (IMWG) updated criteria for the diagnosis of multiple myeloma; (2) age above 18 years old; (3) able to be regularly followed up; (4) life expectancy over 12 months. The exclusion criteria were as follows: (1) relapsed/refractory MM; (2) received stem cell transplantation (SCT), chemotherapy, radiotherapy or other systematic treatments before enrollment; (3) with a history of solid tumors or hematological malignancies other than MM; (4) pregnant/lactating women. In addition, there were 36 healthy bone marrow donors being recruited as controls during the same period. The present study was approved by the Ethics Committee of the Affiliated Hospital of Nantong University, and all participants provided written informed consents.

### Baseline data collection

After enrollment, baseline characteristics were collected from all patients, which included: information of age, gender, immunoglobulin subtype, hemoglobin (Hb), calcium, serum creatinine (Scr), albumin (ALB), Beta-2-microglobulin (β2-MG), Durie-Salmon Stage, the International Staging System (ISS) Stage, bone lesion, lactate dehydrogenase (LDH) and cytogenetics abnormality. Durie-Salmon Stage and ISS Stage were assessed according to the Durie-Salmon Criteria and ISS Criteria respectively [16, 17], and cytogenetics abnormalities were analyzed by fluorescence in situ hybridization (FISH).

### Sample collection and Circ-SMARCA5 detection

Bone marrow samples of all patients were collected prior to initiation of therapy, and bone marrow samples of controls were also obtained after enrollment. Mononuclear cells were separated from bone marrow by gradient density centrifugation, then plasma cells were purified using CD138-coated magnetic beads according to the manufacturer’s instructions (Miltenyi Biotec, Germany) to ensure greater than 90% plasma cell purity. Subsequently, Circ-SMARCA5 expression was determined by quantitative polymerase chain reaction (qPCR) assay.

### Treatment and assessment

All patients received induction therapies after enrollment, and the selections of chemotherapy regimens entirely depended on patients’ willingness and disease conditions. Fourty-eight patients received conventional chemotherapy, 36 patients underwent bortezomib-based regimen and 21 received immunomodulatory drug-based regimen, details were as follows: 26 treated with VAD regimen (doxorubicin/vincristine/dexamethasone); 22 treated with DVD regimen (liposomal doxorubicin/ vincristine/dexamethasone); 36 treated with BCD regimen (bortezomib/cyclophosphamide/dexamethasone), 21 treated with MPT regimen (melphalan/prednisone/thalidomide). After induction treatment, 31 patients underwent autologous SCT, and others received maintenance therapies or repeated induction regimens according to the response to induction treatment. The treatment responses including complete response (CR), very good partial response (VGPR), partial response (PR) were assessed according to the IMWG criteria [18], and the overall response rate (ORR) was defined as the percentage of patients with the achievement of CR, VGPR or PR. All MM patients were regularly followed up to 2017/12/31, and the median follow-up duration was 24.0 months (range: 5.0–36.0 months). Furthermore, progression free survival (PFS) and overall survival (OS) were calculated, which were defined as: PFS, the duration from the treatment to disease progression or death; OS, the duration from the treatment to the death.

### Cells culture

In order to further explore the role of Circ-SMARCA5 in MM etiology, cells experiments were subsequently performed. Wild type MM cell lines (NCI-H929, RPMI8226, U226, OPM2 and JJN3) were purchased from Chinese Academy of Sciences (Beijing, China) or kindly given by Shanghai Jiaotong University (Shanghai, China), and normal plasma cells were isolated from bone marrow of healthy donors using CD138-coated magnetic beads (Miltenyi Biotec, Germany) as normal control. NCI-H929, RPMI8226, U226 and OPM2 cells were cultured in 90% RPMI-1640 medium (Gibco, USA) (containing 1.5 g/L NaHCO_3_, 2.5 g/L glucose and 0.11 g/L sodium pyruvate) supplemented with 10% fetal bovine serum (FBS) (Gibco, USA); JJN3 cells were cultured in 40% DMEM medium (Gibco, USA) and 40% IMEM medium (Gibco, USA) supplemented with 20% FBS (Gibco, USA). All cells were incubated at 37 °C under 95% air and 5% CO_2_ condition.

Measurement of Circ-SMARCA5 in MM cell lines and normal plasma cells.

The qPCR was performed to detect the expression of Circ-SMARCA5 in wild-type MM cell lines: NCI-H929, RPMI8226, U226, OPM2 and JJN3, and normal plasma cells.

### Transfection, groups and followed measurements in RPMI8226 cells

Blank overexpression, Circ-SMARCA5 overexpression, blank shRNA and Circ-SMARCA5 shRNA plasmids were constructed by Shanghai GenePharma Bio-Tech Company (Shanghai, China), and then transfected into RPMI8226 cells as NC (+), Circ (+), NC (−) and Circ (−) groups. Then in each group, qPCR was performed to measure Circ-SMARCA5 expression at 24 h, CCK8 was performed to measure cell proliferation at 0 h, 24 h, 48 h and 72 h, AV/PI was performed to measure cell apoptosis rate at 72 h, and Western Blot was performed to measure apoptotic markers Cleaved Caspase 3 and Bcl-2 expressions at 72 h post transfection. The detailed procedure of each detection was described in the following subsections.

### Validation in NCI-H929 cells

In order to validate the effect of Circ-SMARCA5 on cell proliferation and apoptosis, the transfections of blank overexpression, Circ-SMARCA5 overexpression, blank shRNA and Circ-SMARCA5 shRNA plasmids were repeated in NCI-H929 cells, as well as qPCR at 24 h for detection of Circ-SMARCA5, CCK8 at 0 h, 24 h, 48 h and 72 h for cell proliferation measuement, AV/PI at 72 h for measurement of cell apoptosis, and measurement of apoptotic markers Cleaved Caspase 3 and Bcl-2 expressions at 72 h post transfection by Western Blot.

### Target miRNAs prediction and their measurement

Potential target miRNAs of Circ-SMARCA5 in MM were predicted using Circular RNA Interactome database (https://Circinteractome.nia.nih.gov/) and miRanda database (http://www.microrna.org/microrna/home.do), and miR-561, miR-616 as well as miR-767-5p were selected for validation. The following qPCR was performed to measure their expressions at 24 h post transfection in Control (+), SMARCA5 (+), Control (−) and SMARCA5 (−) groups.

### Regulation of miR-767-5p by circ-SMARCA5 in several MM cell lines

In order to further validate the regulation of miR-767-5p by circ-SMARCA5 in MM, blank overexpression, Circ-SMARCA5 overexpression, blank shRNA and Circ-SMARCA5 shRNA plasmids were transfected into NCI-H929, U226, OPM2 and JJN3 cell lines, and miR-767-5p was detected at 24 h post transfection by qPCR.

### Rescue experiment in RPMI8226 cells

In order to explore the underlying mechanism of Circ-SMARCA5 on regulating cell functions in MM cells, rescue experiment was then conducted. Blank overexpression, miR-767-5p overexpression, Circ-SMARCA5 overexpression, and miR-767-5p overexpression & Circ-SMARCA5 overexpression plasmids were constructed by Shanghai GenePharma Bio-Tech Company (Shanghai, China) and then transfected into RPMI8226 cells as Control (+), miR-767 (+), SMARCA5 (+), and SMARCA5 (+)/miR-767 (+) groups. Then in each group, qPCR was performed to measure miR-767-5p and Circ-SMARCA5 expressions at 24 h, CCK8 was performed to measure cell proliferation at 0 h, 24 h, 48 h and 72 h, AV/PI was performed to measure cell apoptosis rate at 72 h, and Western Blot was performed to measure apoptotic markers Cleaved Caspase 3 and Bcl-2 expressions at 72 h post transfection. The detailed procedure of each detection was described in the following subsections.

### Luciferase reporter assay

In order to further validate the interaction between Circ-SMARCA5 and miR-767-5p, luciferase reporter assay was subsequently conducted as follows: (1) the RPMI8226 cells were digested and inoculated on a culture plate and left in 5% CO2, humidity saturated incubator (37 °C) over night. (2) When cell density exceeded 70%, co-transfecting the cells with Circ-SMARCA5 wild type/ mutant type luciferase reporter plasmids and miR-767-5p overexpression/control plasmids. Reporter plasmids and overexpression plasmids were constructed by Shanghai GenePharma Bio-Tech Company (Shanghai, China). (3) At 24 h after transfection, the culture solution was removed and the cells were washed with cold PBS. (4) Add 350 μl pre-cooled harvest buffer into each culture dish and the cells were lysed at 4 °C or on ice for 10 mins. (5) Centrifuge for 5 s at 12,000 rpm. Transfering the supernatant to a new tube and discard the pelleted cell debris. (6) 5 ml microcentrifuge tubes were prepared by adding 100 μl of reactant solution of ATP buffer mixed with luciferin buffer (in ratio of 1:3.6) to each tube. (7) 100 μl of cell lysate supernatant from step 5 was added into each of the microcentrifuges prepared in step 6, blending quickly and read the absorbance value on a luminometer. (8) Ensure to read the absorbance value of all samples with the same procedure, then measured the activity of LacZ from the rest of the cell lysate and used it as an internal reference to correct the readings of luciferase.

### qPCR

Total RNA was extracted using TRIzol reagent (Invitrogen, USA) and then applied for the synthesis of cDNA using PrimeScript™ RT reagent Kit (with random primers for Circ-SMARCA5) (TAKARA, Japan). Following that, cDNA was used for qPCR with SYBR Green kit (TaKaRa, Japan). Amplification of PCR was conducted under the following condition: 95 °C for 3 min, 40 cycles of 95 °C for 5 s, 61 °C for 10 s, and then 72 °C for 30s. The result was calculated using 2^-ΔΔCt^ method and glyceraldehyde-3-phosphate dehydrogenase (GAPDH) was used as an internal reference. The primers used in qPCR were listed in Additional file 1: Table S1.

### Western blot

After lysing with RIPA lysis buffer (Thermo Scientific, USA), RPMI8226 cells were centrifuged, then total protein concentration was measured using BCA™ protein assay kit (Pierce, USA). Electrophoresis was used for separating proteins on sodium dodecyl sulfate-polyacrylamide (SDS) gel. Subsequently, PVDF membrane (Millipore, USA) was used to transfer proteins and then blocked with 5% non-fat dried milk in PBST for 1 h at room temperature. After incubation with primary antibodies, the PVDF membrane was finally incubated with the secondary antibody. ECL advanced Western blot analysis detection kit (BD, USA) was then used to visualize the bands indicating the abundance of proteins. The grey intensity was measured using ImageJ (NIH, USA). The antibodies used in Western Blot were listed in Additional file 2: Table S2.

### CCK8

Ten ul CCK-8 (Dojindo, Japan) and 90 ul medium were added to each group of RPMI8226 cells, then the cells were incubated at 37 °C under 95% air + 5% CO2. Cell proliferation ability was presented by optical density (OD) value, which was measured using a microplate reader (BioTek, USA).

### Av/pi

RPMI8226 cells were digested using pancreatin and washed with phosphate buffer solution, then suspended in blinding buffer (100 ul). 10 ul AV (Invitrogen, USA) was subsequently added and the cells were left on ice for 15 min in dark. Following that, 5 ul PI (Invitrogen, USA) was added and the apoptosis rate was analyzed by flow cytometry (FCM) (Becton Dickinson, USA).

### Statistics

Statistical analysis was performed using SPSS 22.0 software (IBM, USA), and figures were made with the use of GraphPad Prism 7.00 software (GraphPad Software Inc., USA). Count data were expressed as count (percentage); Continuous data were described as mean ± standard deviation (clinical data) or mean ± standard error (experimental data) if normally distributed, and as median (25th–75th quantiles) if not normally distributed. Comparison was determined by Chi-square test, t test or Wilcoxon rank sum test. Receiver operating characteristic (ROC) curve was used to evaluate the ability of Circ-SMARCA5 relative expression to discriminate between MM patients and controls. Survival curves were made using the Kaplan-Meier method and significant differences between the curves were determined by log-rank test. Univariate and multivariate logistic regression analyses with Forward Stepwise (Conditional) method were performed to assess the predictive factors of CR; univariate and multivariate Cox’s proportional hazards regression analyses with Forward Stepwise (Conditional) method were performed to determine the factors affecting PFS and OS. *P* value < 0.05 was considered as significant.

## Results

### Patients’ baseline characteristics

One hundred-and five MM patients with mean age 59.8 ± 9.1 years were enrolled, and among which 65 (61.9%) were males and 40 (38.1%) were females. As to the disease staging, the numbers of patients with Durie-Salmon stage I, II, and III were 4 (3.8%), 49 (46.7%) and 52 (49.5%) respectively. Additionally, there were 24 (22.9%), 36 (34.3%) and 45 (42.8%) patients with ISS stage I, II, and III respectively. Other detailed characteristics were listed in Table 1.

**Table 1:** Baseline characteristics of MM patients

Parameters	MM patients (*N* = 105)
Age (years)	59.8 ± 9.1
Gender (n/%)	
Male	65 (61.9)
Female	40 (38.1)
Immunoglobulin subtype (n/%)	
IgG	59 (56.1)
IgA	24 (22.9)
Bence-Jones protein	19 (18.1)
IgD	2 (1.9)
IgM	1 (1.0)
Hb (g/dL)	10.0 ± 2.4
Calcium (mg/dL)	10.0 ± 1.8
Scr (mg/dL)	1.6 ± 0.5
ALB (mg/dL)	3.9 ± 0.7
β2-MG (mg/L)	4.8 (2.8–8.4)
LDH (U/L)	191.7 (169.0–228.3)
Durie-Salmon stage (n/%)	
I	4 (3.8)
II	49 (46.7)
III	52 (49.5)
ISS stage (n/%)	
I	24 (22.9)
II	36 (34.3)
III	45 (42.8)
Bone lesion (n/%)	75 (71.4)
Cytogenetics abnormality (n/%)	
t (14; 16) translocation	15 (14.3)
Del (17p)	14 (13.3)
t (4; 14) translocation	10 (9.5)

Data were presented as mean value ± standard deviation, count (percentage) or median (25th–75th quantiles)

*MM* multiple myeloma, *Ig* immunoglobulin, *Hb* hemoglobin, *Scr* serum creatinine, *ALB* albumin, *β2-MG* Beta-2-microglobulin, *LDH* lactate dehydrogenase, *ISS* International Staging System

### Circ-SMARCA5 relative expression in MM patients

**Fig. 1:**
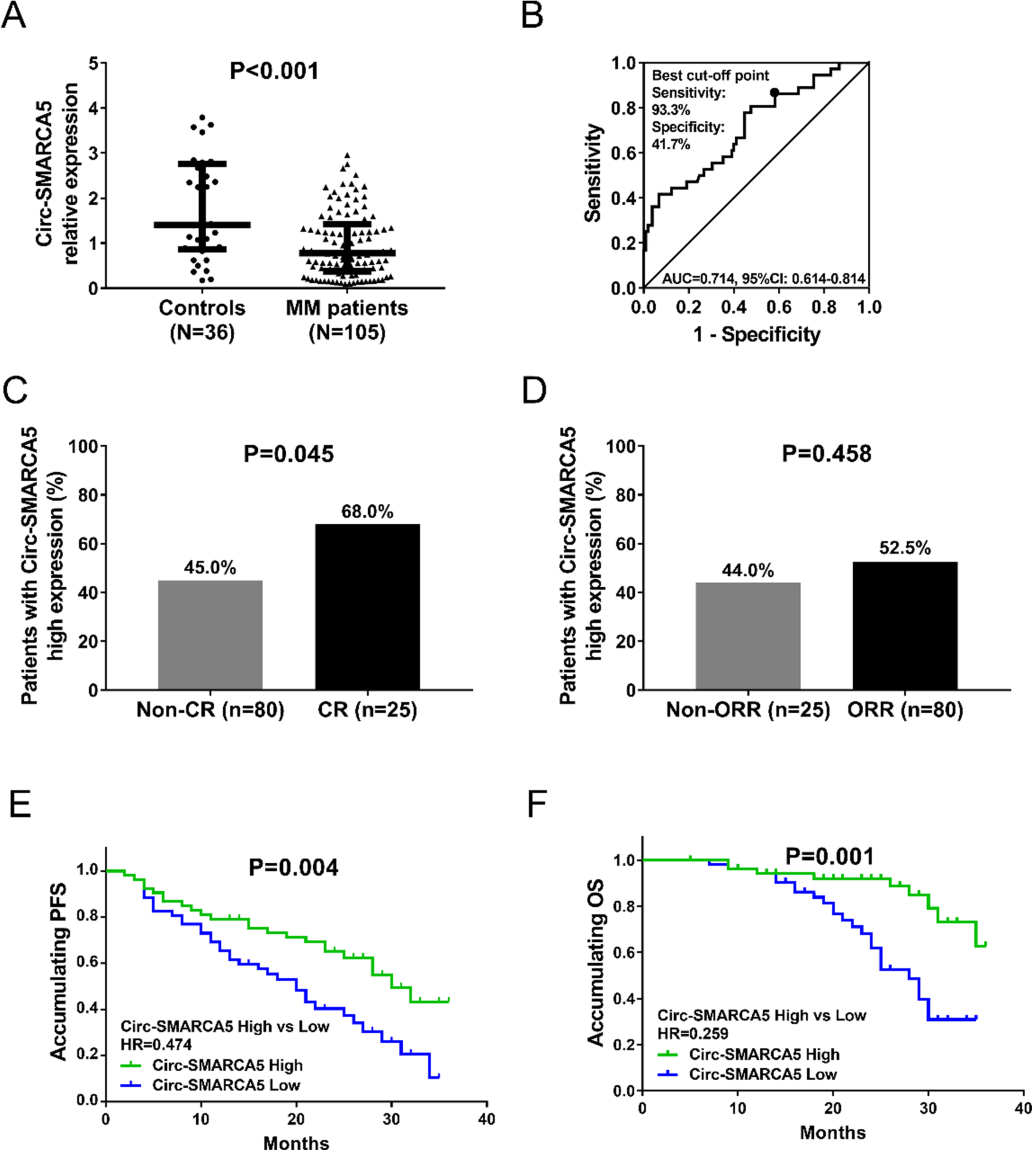
**a-b:** Circ-SMARCA5 relative expression in MM patients. Wilcoxon rank sum test was used to compare Circ-SMARCA5 relative expression between MM patients and controls, and the ROC curve was used to evaluate the ability of Circ-SMARCA5 relative expression to discriminate between MM patients and controls. **c- d**: Association of Circ-SMARCA5 expression with treatment response in MM patients. The cutoff value of Circ-SMARCA5 was the median value of Circ-SMARCA5 in MM patients. Comparison between two groups was performed using the Chi-square test. **e- f**: Comparison of PFS and OS between Circ-SMARCA5 high expression and low expression groups. The cutoff value of Circ-SMARCA5 was the median value of Circ-SMARCA5 in MM patients. Survival curves were made using the Kaplan-Meier method and differences between the curves were determined by log-rank test. *P* < 0.05 was considered significant. PFS, progression free survival; OS, overall survival; Circ-SMARCA5, Circular RNA SWI/SNF-related matrix-associated actin-dependent regulator of chromatin subfamily A member 5; ROC, Receiver operating characteristic. CR, complete response; ORR, objective response rate

The median Circ-AMARCA5 relative expression was 0.778 (0.377–1.421) in MM patients, which was lower than that in control group (1.407 (0.864–2.763)) (*P* < 0.001) (Fig. 1a). ROC curve illustrated that Circ-AMARCA5 was of good value in distinguishing MM patients from the controls with area under curve (AUC) of 0.714 (95%CI: 0.614–0.814). The sensitivity and specificity at the best cut-off point (the point where the largest sum of sensitivity and specificity occurred) were 93.3 and 41.7% respectively and the cut-off value of Circ-AMARCA5 relative expression was 2.242 (Fig. 1b).

### Association between Circ-SMARCA5 relative expression and patients’ baseline characteristics

All patients were divided into high expression and low expression groups depending on whether their Circ-SMARCA5 relative expression was greater than the median value in MM patients or not, which showed that Circ-SMARCA5 high expression was correlated with lower β2-MG level (*P* = 0.001) as well as less advanced ISS stage (*P* < 0.001) (Table 2).

**Table 2:** Correlation of Circ-SMARCA5 relative expression with patients’ characteristics

Characteristics	Circ-SMARCA5 relative expression		*p* value
	High	Low	
Age (n/%)			0.143
< 60 years	23 (43.4)	30 (56.6)	
≥ 60 years	30 (57.7)	22 (42.3)	
Gender (n/%)			0.632
Male	34 (52.3)	31 (47.7)	
Female	19 (47.5)	21 (52.5)	
Immunoglobulin subtypes (n/%)			0.537
IgA	14 (58.3)	10 (41.7)	
IgG	31 (52.5)	28 (47.5)	
IgD	1 (50.0)	1 (50.0)	
Bence-Jones protein	7 (36.8)	12 (63.2)	
IgM	0 (0.0)	1 (100.0)	
Hb (n/%)			0.494
< 10 g/dL	28 (53.8)	24 (46.2)	
≥ 10 g/dL	25 (47.12)	28 (52.8)	
Calcium (n/%)			0.346
< 11.5 mg/dL	41 (53.2)	36 (46.8)	
≥ 11.5 mg/dL	12 (42.9)	16 (57.1)	
Scr (n/%)			0.604
< 2 mg/dL	42 (51.9)	39 (48.1)	
≥ 2 mg/dL	11 (45.8)	13 (54.2)	
ALB (n/%)			0.782
< 3.5 mg/dL	16 (48.5)	17 (51.5)	
≥ 3.5 mg/dL	37 (51.4)	35 (48.6)	
β2-MG (n/%)			**0.001**
< 5.5 mg/L	39 (65.0)	21 (35.0)	
≥ 5.5 mg/L	14 (31.3)	31 (68.9)	
LDH (n/%)			0.361
< 220 U/L	39 (53.4)	34 (46.6)	
≥ 220 U/L	14 (43.8)	18 (56.3)	
Durie-Salmon stage (n/%)			0.650
I	3 (75.0)	1 (25.0)	
II	22 (44.9)	27 (55.1)	
III	28 (53.8)	24 (46.2)	
ISS stage (n/%)			**< 0.001**
I	18 (75.0)	6 (25.0)	
II	21 (58.3)	15 (41.7)	
III	14 (31.1)	31 (68.9)	
Bone lesion (n/%)			0.174
Yes	41 (54.7)	34 (45.3)	
No	12 (40.0)	18 (60.0)	
Cytogenetics abnormalities (n/%)			
t (14; 16) translocation			0.056
Yes	11 (73.3)	4 (26.7)	
No	42 (46.7)	48 (53.3)	
t (4; 14) translocation			0.486
Yes	4 (40.0)	6 (60.0)	
No	49 (51.6)	46 (48.4)	
Del (17p)			0.235
Yes	5 (35.7)	9 (64.3)	
No	48 (52.7)	43 (47.3)	

Data were presented as count (percentage). Patients’ circ-SMARCA5 relative expression classified as high if greater than the median, and low if less than the median value. Comparison was determined by Chi-square test or Wilcoxon rank sum test. *p* value < 0.05 was considered significant (in bold)

*Ig* immunoglobulin, *Hb* hemoglobin, *Scr* serum creatinine, *ALB* albumin, *β2-MG* Beta-2-microglobulin, *LDH* lactate dehydrogenase, *ISS* International Staging System

Correlation between treatment response of MM patients and Circ-SMARCA5 expression.

There were 25 patients who achieved CR and 80 patients who did not. The expression of Circ-SMARCA5 was higher in CR patients compared to non-CR patients (*P* = 0.045) (Fig. 1c). As for ORR, 80 patients achieved ORR but 25 patients did not, and Circ-SMARCA5 expression was similar between ORR patients and non-ORR patients (*P* = 0.458) (Fig. 1d).

### Factors influencing CR in MM patients

Univariate logistic regression revealed that Circ-SMARCA5 (high vs low) (OR = 2.597, *P* = 0.049) was correlated with higher CR, whereas β2-MG (≥5.5 mg/L vs < 5.5 mg/L) (OR = 0.332, *P* = 0.034) and ISS stage (III vs I&II) (OR = 0.332, *P* = 0.034) were associated with lower CR (Table 3). Further multivariate logistic regression with Forward stepwise (Conditional) method illustrated that ISS stage (III vs I&II) independently predicted lower CR (OR = 0.332, *P* = 0.034) in MM patients.

**Table 3:** Factors affecting CR by logistic regression analysis

Factors	Logistic regression model			
	p value	OR	95%CI	
			Lower	Higher
Univariate logistic regression				
Circ-SMARCA5 (high vs low)	**0.049**	2.597	1.006	6.707
Age (≥60 years vs < 60 years)	0.278	0.603	0.242	1.502
Gender (male vs female)	0.822	0.900	0.359	2.257
Immunoglobulin subtype				
IgG vs others	0.369	1.530	0.605	3.867
IgA vs others	0.150	0.383	0.104	1.413
IgM vs others	1.000	0.000	0.000	–
IgD vs others	0.406	3.292	0.198	54.631
Bence-Jones protein vs others	0.777	1.179	0.378	3.674
Hb (≥10 g/dL vs < 10 g/dL)	0.101	0.460	0.182	1.164
Calcium ≥11.5 mg/dL vs < 11.5 mg/dL)	0.391	0.620	0.208	1.848
Scr (≥2 mg/dL vs < 2 mg/dL)	0.484	1.441	0.517	4.014
ALB (≥3.5 mg/dL vs < 3.5 mg/dL)	0.362	1.613	0.577	4.511
β2-MG (≥5.5 mg/L vs < 5.5 mg/L)	**0.034**	0.332	0.120	0.918
LDH (≥220 U/L vs < 220 U/L)	0.198	0.491	0.166	1.451
Durie-Salmon stage (III vs I&II)	0.233	1.743	0.700	4.343
ISS stage (III vs I&II)	**0.034**	0.332	0.120	0.918
Bone lesion (yes vs no)	0.942	1.038	0.382	2.816
Cytogenetics abnormality				
t (4; 14) translocation (yes vs no)	0.767	0.783	0.155	3.951
t (14; 16) translocation (yes vs no)	0.709	0.773	0.200	2.991
Del (17p) (yes vs no)	0.377	0.493	0.103	2.368
Multivariate logistic regression with Forward Stepwise (Conditional) method				
ISS stage (III vs I&II)	**0.034**	0.332	0.120	0.918

Factors affecting CR were determined by univariate and multivariate logistic regression analyses, and the multivariate logistic regression analysis was performed with Forward Stepwise (Conditional) method. p value < 0.05 was considered significant (in bold)

*CR* complete remission, *OR* odds ratio, *CI* confidence interval, *Ig* immunoglobulin, *Hb* hemoglobin, *Scr* serum creatinine, *ALB* albumin, *β2-MG* Beta-2-microglobulin, *LDH* lactate dehydrogenase, *ISS* International Staging System

Comparison of survivals between Circ-SMARCA5 high expression and low expression groups.

Accumulating PFS was higher in Circ-SMARCA5 high expression group compared with Circ-SMARCA5 low expression group (*P* = 0.004) (Fig. 1e). Also, accumulating OS was longer in Circ-SMARCA5 high expression group compared with Circ-SMARCA5 low expression group (*P* = 0.001) (Fig. 1f).

### Factors influencing PFS in MM patients

Univariate Cox’s regression disclosed that Circ-SMARCA5 (high vs low) (HR = 0.474, *P* = 0.006) was correlated with better PFS, while Scr (≥2 mg/dL vs < 2 mg/dL) (HR = 2.261, *P* = 0.005), β2-MG (≥5.5 mg/L vs < 5.5 mg/L) (HR = 22.970, *P* < 0.001) and ISS stage (III vs I&II)) (HR = 22.970, *P* < 0.001) were associated with worse PFS (Table 4). Additionally, multivariate Cox’s regression with Forward Stepwise (Conditional) method illustrated that Durie-Salmon stage (III vs I&II) (HR = 2.350, *P* = 0.002) and ISS stage (III vs I&II) (HR = 32.620, *P* < 0.001) independently predicted worse PFS in MM patients.

**Table 4:** Factors affecting PFS by Cox’s proportional hazards regression analysis

Items	Cox’s regression model			
	*p* value	HR	95%CI	
			Lower	Higher
Univariate Cox’s regression				
Circ-SMARCA5 (high vs low)	**0.006**	0.474	0.278	0.806
Age (≥60 years vs < 60 years)	0.390	1.255	0.748	2.106
Gender (male vs female)	0.122	0.663	0.393	1.116
Immunoglobulin subtype				
IgG vs others	0.583	0.865	0.516	1.450
IgA vs others	0.423	1.272	0.706	2.290
IgM vs others	0.720	1.436	0.198	10.423
IgD vs others	0.873	0.851	0.117	6.178
Bence-Jones protein vs others	0.847	0.935	0.472	1.850
Hb (≥10 g/dL vs < 10 g/dL)	0.908	0.970	0.578	1.626
Calcium ≥11.5 mg/dL vs < 11.5 mg/dL)	0.749	0.906	0.497	1.655
Scr (≥2 mg/dL vs < 2 mg/dL)	**0.005**	2.261	1.282	3.990
ALB (≥3.5 mg/dL vs < 3.5 mg/dL)	0.792	1.080	0.612	1.905
β2-MG (≥5.5 mg/L vs < 5.5 mg/L)	**< 0.001**	22.970	9.519	55.426
LDH (≥220 U/L vs < 220 U/L)	0.480	1.219	0.703	2.113
Durie-Salmon stage (III vs I&II)	0.320	1.302	0.774	2.191
ISS stage (III vs I&II)	**< 0.001**	22.970	9.519	55.426
Bone lesion (yes vs no)	0.239	0.705	0.393	1.262
Cytogenetics abnormality				
t (4; 14) translocation (yes vs no)	0.947	0.972	0.413	2.286
t (14; 16) translocation (yes vs no)	0.589	0.804	0.365	1.773
Del (17p) (yes vs no)	0.453	1.313	0.644	2.676
Multivariate Cox’s regression with Forward Stepwise (Conditional) method				
Durie-Salmon stage (III vs I&II)	**0.002**	2.350	1.363	4.053
ISS stage (III vs I&II)	**< 0.001**	32.620	12.739	83.528

Factors affecting PFS were determined by univariate and multivariate Cox’s proportional hazards regression analyses, and the multivariate Cox’s proportional hazards regression analysis was performed with Forward Stepwise (Conditional) method. p value < 0.05 was considered significant (in bold)

*PFS* progression free survival, *HR* hazard ratio, *CI* confidence interval, *Ig* immunoglobulin, *Hb* hemoglobin, *Scr* serum creatinine, *ALB* albumin, *β2-MG* Beta-2-microglobulin, *LDH* lactate dehydrogenase, *ISS* International Staging System

### Factors influencing OS in MM patients

Univariate Cox’s regression revealed that Circ-SMARCA5 (high vs low) (HR = 0.259, *P* = 0.001) was correlated with better OS, whereas β2-MG (≥5.5 mg/L vs < 5.5 mg/L) (HR = 108.139, *P* < 0.001) and ISS stage (III vs I&II)) (HR = 108.139, *P* < 0.001) were risk factors for shorter OS (Table 5). Multivariate Cox’s regression with Forward Stepwise (Conditional) method was further performed, which exhibited that Circ-SMARCA5 (high vs low) (HR = 0.345, *P* = 0.038) independently predicted longer OS, while Durie-Salmon stage (III vs I&II) (HR = 2.586, *P* = 0.015) and ISS stage (III vs I&II) (HR = 148.290, *P* < 0.001) independently predicted shorter OS in MM patients.

**Table 5:** Factors affecting OS by Cox’s proportional hazards regression analysis

Items	Cox’s regression model			
	*p* value	HR	95%CI	
			Lower	Higher
Univariate Cox’s regression				
Circ-SMARCA5 (high vs low)	**0.001**	0.259	0.119	0.565
Age (≥60 years vs < 60 years)	0.545	1.238	0.620	2.472
Gender (male vs female)	0.074	0.535	0.269	1.064
Immunoglobulin subtype				
IgG vs others	0.805	1.091	0.546	2.179
IgA vs others	0.936	0.966	0.418	2.231
IgM vs others	0.609	0.048	0.000	5345.739
IgD vs others	0.837	1.233	0.168	9.065
Bence-Jones protein vs others	0.959	0.977	0.403	2.369
Hb (≥10 g/dL vs < 10 g/dL)	0.616	1.193	0.600	2.371
Calcium ≥11.5 mg/dL vs < 11.5 mg/dL)	0.507	1.286	0.611	2.705
Scr (≥2 mg/dL vs < 2 mg/dL)	0.858	1.085	0.445	2.645
ALB (≥3.5 mg/dL vs < 3.5 mg/dL)	0.887	0.947	0.448	2.001
β2-MG (≥5.5 mg/L vs < 5.5 mg/L)	**< 0.001**	108.139	14.142	826.884
LDH (≥220 U/L vs < 220 U/L)	0.385	1.370	0.673	2.789
Durie-Salmon stage (III vs I&II)	0.287	1.465	0.725	2.959
ISS stage (III vs I&II)	**< 0.001**	108.139	14.142	826.884
Bone lesion (yes vs no)	0.687	0.854	0.395	1.842
Cytogenetics abnormality				
t (4; 14) translocation (yes vs no)	0.747	0.822	0.250	2.706
t (14; 16) translocation (yes vs no)	0.465	0.642	0.196	2.106
Del (17p) (yes vs no)	0.575	1.314	0.506	3.408
Multivariate Cox’s regression with Forward Stepwise (Conditional) method				
Circ-SMARCA5 (high vs low)	**0.038**	0.345	0.127	0.941
Durie-Salmon stage (III vs I&II)	**0.015**	2.586	1.206	5.546
ISS stage (III vs I&II)	**< 0.001**	148.290	17.354	1267.115

Factors affecting OS were determined by univariate and multivariate Cox’s proportional hazards regression analyses, and the multivariate Cox’s proportional hazards regression analysis was performed with Forward Stepwise (Conditional) method. p value < 0.05 was considered significant (in bold)

*OS* overall survival, *HR* hazard ratio, *CI* confidence interval, *Ig* immunoglobulin, *Hb* hemoglobin, *Scr* serum creatinine, *ALB* albumin, *β2-MG* Beta-2-microglobulin, *LDH* lactate dehydrogenase, *ISS* International Staging System

### Circ-SMARCA5 relative expression in MM cell lines and normal plasma cells

**Fig. 2:**
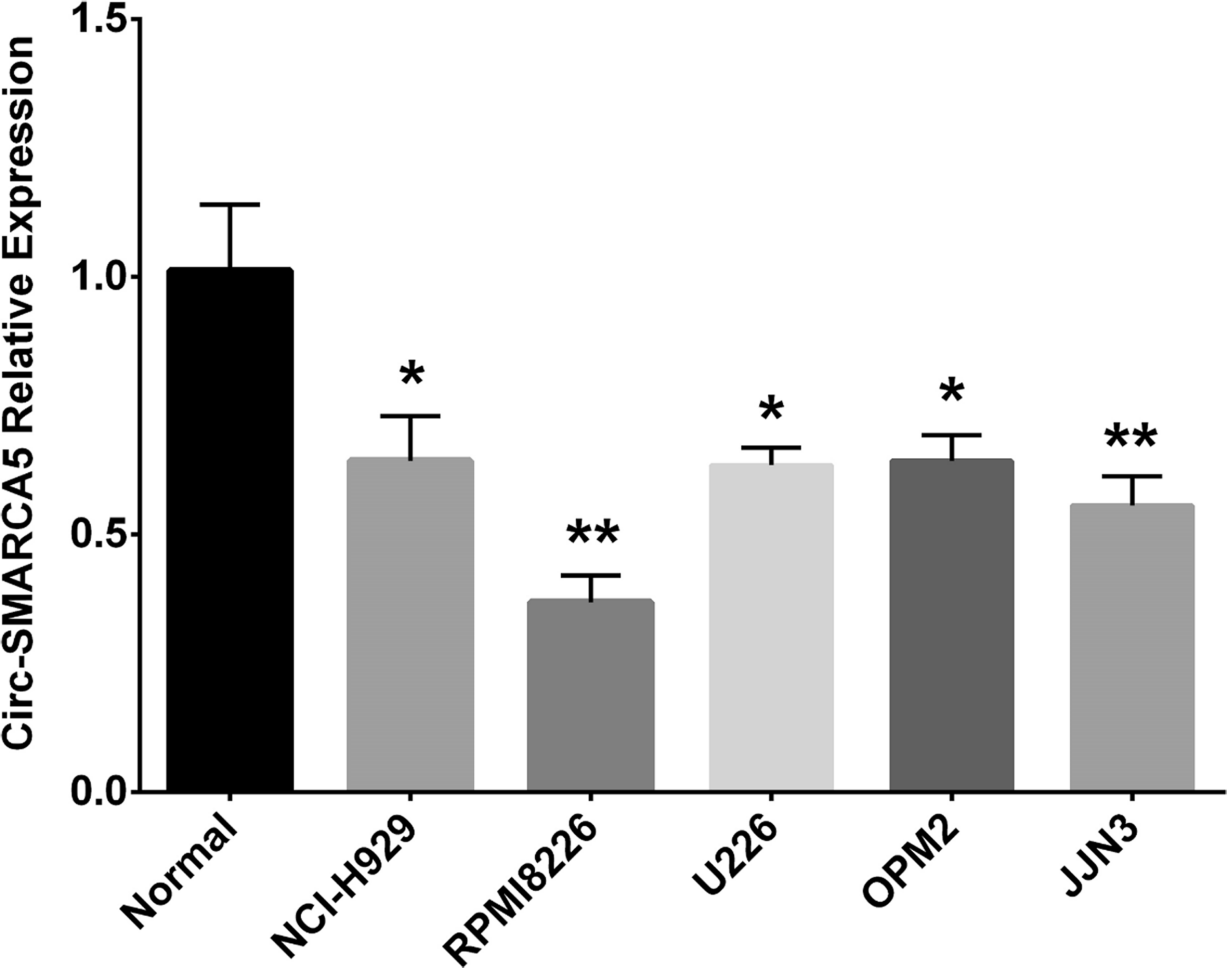
Comparing Circ-SMARCA5 expression in MM cell lines and control cells. Circ-SMARCA5 expression was decreased in MM cell lines including NCI-H929, RPMI8226, U226, OPM2 and JJN3 compared with control cells, and RPMI8226 cells presented the lowest Circ-SMARCA5 expression. Comparison of Circ-SMARCA5 expression between control cells and each MM cell line was performed by t test, and *P* < 0.05 was considered significant. *P < 0.05, **P < 0.01. Circ-SMARCA5, Circular RNA SWI/SNF-related matrix-associated actin-dependent regulator of chromatin subfamily A member 5

In order to get a deeper understanding of the underlying mechanism of Circ-SMARCA5 in MM, in vitro experiments were performed to investigate the effect of Circ-SMARCA5 on cell activities as well as its possible target in MM cells. Circ-SMARCA5 relative expression in MM cell lines and normal plasma cells was detected by qPCR, which revealed that Circ-SMARCA5 was downregulated in MM cell lines including NCI-H929 (*P* < 0.05), RPMI8226 (*P* < 0.01), U226 (*P* < 0.05), OPM2 (*P* < 0.05) and JJN3 (*P* < 0.01) compared to normal plasma cells (Fig. 2). The lowest expression of Circ-SMARCA5 was presented in RPMI8226 cells, thus, RPMI8226 cells were chosen to be applied in the following experiments.

### Circ-SMARCA5 relative expression after transfection

**Fig. 3:**
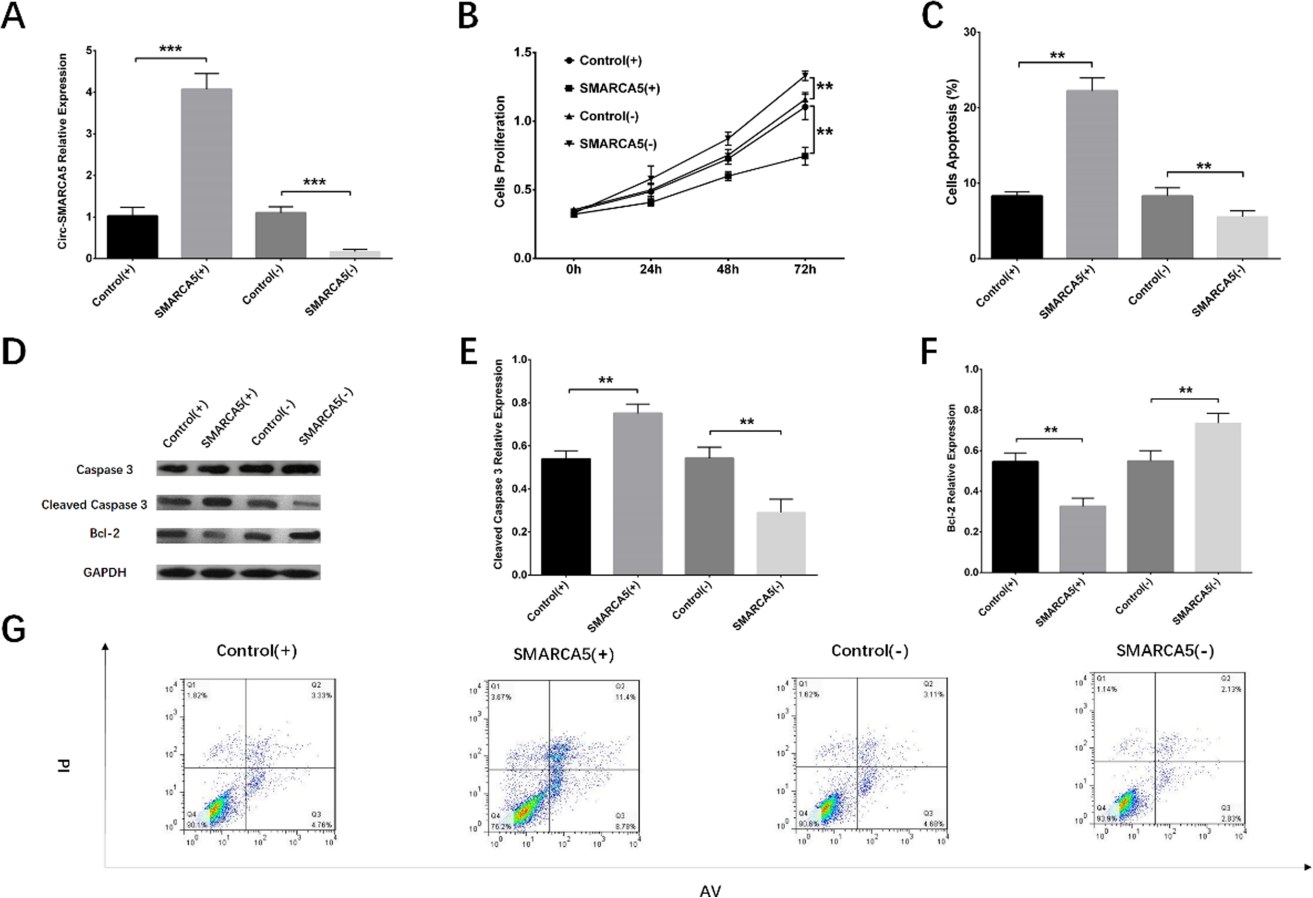
**a:** Circ-SMARCA5 relative expression at 24 h after transfection. Circ-SMARCA5 expression was increased in SMARCA5 (+) group compared to Control (+) group but decreased in SMARCA5 (−) group compared with Control (−) group. Comparison of Circ-SMARCA5 expression between two groups was performed by t test. ***P < 0.001. **b**-**g:** The effects of Circ-SMARCA5 on proliferation and apoptosis of RPMI8226 cells. Cell proliferation was reduced in SMARCA5 (+) group compared with Control (+) group but promoted in SMARCA5 (−) group compared with Control (−) group (**b**). Cell apoptosis rate was increased in SMARCA5 (+) group compared with Control (+) group but decreased in SMARCA5 (−) group compared with Control (−) group (**c**, **g**). The expression of cell apoptotic marker Cleaved Caspase 3 was elevated in SMARCA5 (+) group compared with Control (+) group but lowered in SMARCA5 (−) group compared with Control (−) group, and for Bcl-2, its expression was reduced in SMARCA5 (+) group compared with Control (+) group but raised in SMARCA5 (−) group compared with Control (−) group (**d**, **e**, **f**). Comparisons of cell proliferation ability and apoptosis rate were by t test, and *P* < 0.05 was considered significant. **P < 0.01. Round dot represented Control (+) group, square represented SMARCA5 (+) group, regular triangle represented Control (−) group, inverted triangle represented SMARCA5 (−) group. Circ-SMARCA5, Circular RNA SWI/SNF-related matrix-associated actin-dependent regulator of chromatin subfamily A member 5; Control (+), blank overexpression; SMARCA5 (+), Circ-SMARCA5 overexpression; Control (−), blank shRNA; SMARCA5 (−), Circ-SMARCA5 shRNA

To verify the transfection effects of plasmids, we used qPCR to detect the expression of Circ-SMARCA5 in RPMI8226 cells at 24 h after transfection, and the data showed that Circ-SMARCA5 was upregulated in SMARCA5 (+) group compared to Control (+) group (*P* < 0.001) but downregulated in SMARCA5 (−) group compared with Control (−) group (*P* < 0.001), which indicated successful transfection (Fig. 3a).

### Effect of Circ-SMARCA5 on cell proliferation and apoptosis

**Fig. 4:**
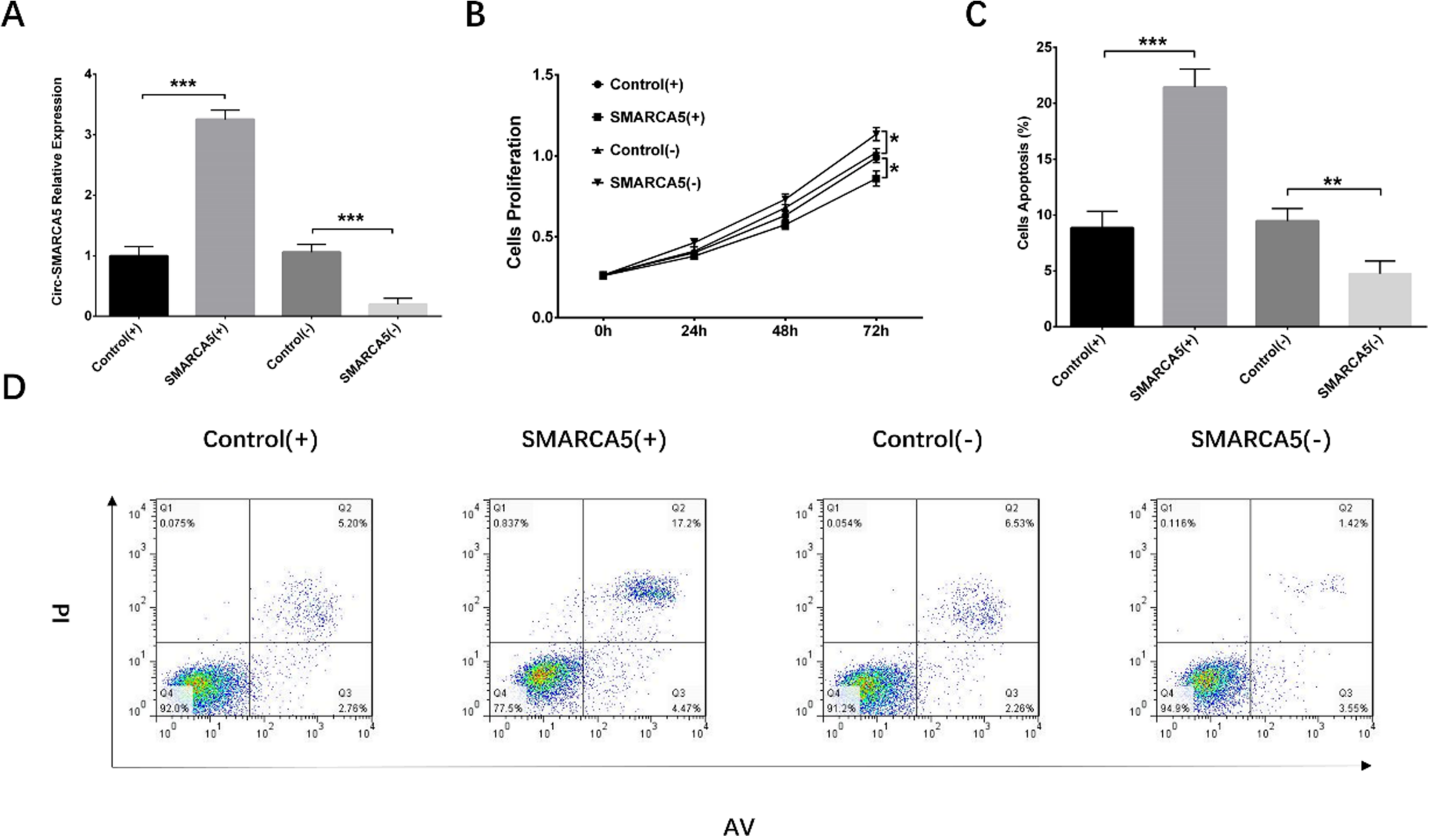
The effects of Circ-SMARCA5 on proliferation and apoptosis of NCI-H929 cells. Circ-SMARCA5 expression was increased in SMARCA5 (+) group compared to Control (+) group but decreased in SMARCA5 (−) group compared with Control (−) group (**a**). Cell proliferation was reduced in SMARCA5 (+) group compared with Control (+) group but promoted in SMARCA5 (−) group compared with Control (−) group (**b**). Cell apoptosis rate was increased in SMARCA5 (+) group compared with Control (+) group but decreased in SMARCA5 (−) group compared with Control (−) group (**c**, **d**). Comparisons of Circ-SMARCA5 expression, cell proliferation ability and apoptosis rate were by t test, and *P* < 0.05 was considered significant. *P < 0.05, **P < 0.01, ***P < 0.001. Round dot represented Control (+) group, square represented SMARCA5 (+) group, regular triangle represented Control (−) group, inverted triangle represented SMARCA5 (−) group. Circ-SMARCA5, Circular RNA SWI/SNF-related matrix-associated actin-dependent regulator of chromatin subfamily A member 5; Control (+), blank overexpression; SMARCA5 (+), Circ-SMARCA5 overexpression; Control (−), blank shRNA; SMARCA5 (−), Circ-SMARCA5 shRNA

Cell proliferation ability was reduced in SMARCA5 (+) group compared with Control (+) group (*P* < 0.01) but promoted in SMARCA5 (−) group compared with Control (−) group (*P* < 0.01) at 72 h after transfection (Fig. 3b). As for cell apoptosis rate, it was increased in SMARCA5 (+) group compared with Control (+) group (*P* < 0.01) but decreased in SMARCA5 (−) group compared with Control (−) group (*P* < 0.01) at 72 h after transfection (Fig. 3c, g). Western blot assay visualized that cells apoptotic marker cleaved caspase 3 was elevated in SMARCA5 (+) group compared with Control (+) group but reduced in SMARCA5 (−) group compared with Control (−) group, whereas Bcl-2 was decreased in SMARCA5 (+) group compared with Control (+) group but increased in SMARCA5 (−) group compared with Control (−) group (Fig. 3d, e, f). To validate this, the above experiments were repeated in NCI-H929 cells, and similar results were observed (Fig. 4).

### Effect of Circ-SMARCA5 on regulating miR-561, miR-616 and miR-767-5p

**Fig. 5:**
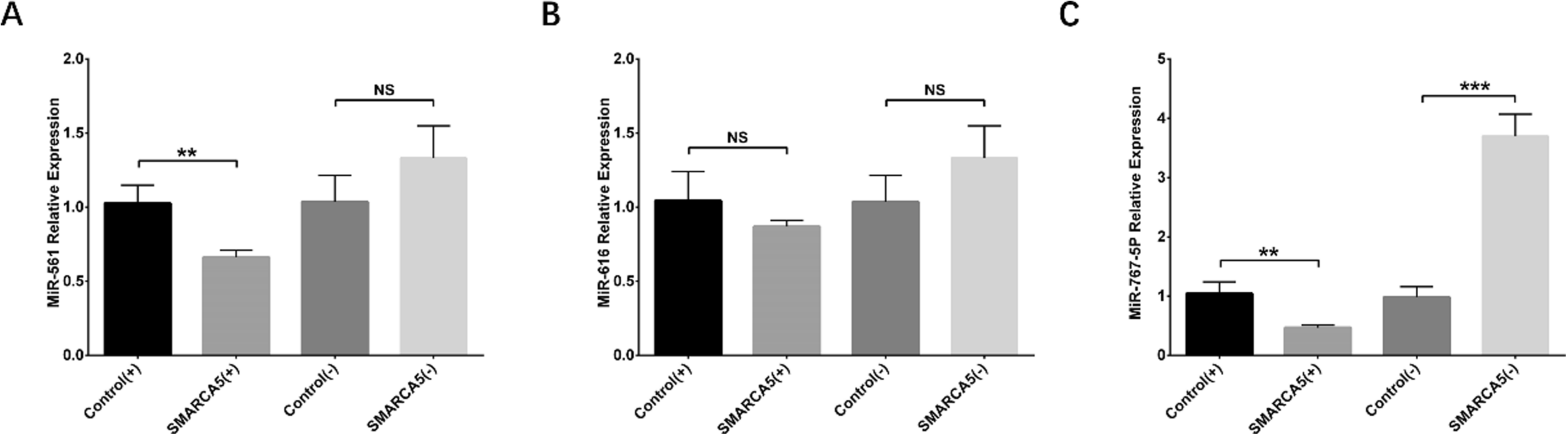
Expression of miR-561, miR-616 and miR-767-5p after transfection. At 72 h after transfection in RPMI8226 cells, miR-561 expression was reduced in SMARCA5 (+) group compared with Control (+) group but similar in SMARCA5 (−) group compared with Control (−) group (**a**). No difference in expression of miR-616 was observed between SMARCA5 (+) and Control (+) groups, or between SMARCA5 (−) and Control (−) groups (**b**). As for miR-767-5p, its expression was reduced in SMARCA5 (+) group compared with NC (+) group but raised in SMARCA5 (−) group compared with Control (−) group. Comparisons of miRNA expressions were performed using t test and *P* < 0.05 was considered significant. **P < 0.01, ***P < 0.001, NS, non-significant. Control (+), blank overexpression; SMARCA5 (+), Circ-SMARCA5 overexpression; Control (−), blank shRNA; SMARCA5 (−), Circ-SMARCA5 shRNA; miR, micro RNA

Potential target miRNAs of Circ-SMARCA5 in MM were predicted using Circular RNA Interactome database and miRanda database, and miR-561, miR-616 as well as miR-767-5p were selected for validation. The qPCR was performed to measure their expressions at 24 h post transfection in Control (+), SMARCA5 (+), Control (−) and SMARCA5 (−) groups, which observed that miR-561 expression was reduced in SMARCA5 (+) group compared with Control (+) group (*P* < 0.01) but similar between SMARCA5 (−) group and Control (−) group (*P* > 0.05) (Fig. 5a). There was no difference in miR-616 expression between SMARCA5 (+) group and Control (+) group (*P* > 0.05) or between SMARCA5 (−) group and Control (−) group (*P* > 0.05) (Fig. 5b). As for miR-767-5p, its expression was decreased in SMARCA5 (+) group compared with Control (+) group (*P* < 0.01) and increased in SMARCA5 (−) group compared with Control (−) group (*P* < 0.001) (Fig. 5c). Besides, we further validated the regulation of miR-767-5p by circ-SMARCA5 in NCI-H929, U226, OPM2 and JJN3 cells, which observed that circ-SMARCA5 negatively regulated miR-767-5p in NCI-H929, U226 and OPM2 significantly (all *P* < 0.01) (Additional file 3: Figure S1A-C), while slightly regulated miR-767-5p in JJN3 cells (all *P* < 0.05) (Additional file 3: Figure S1D).

### Interaction between Circ-SMARCA5 and miR-767-5p in the rescue experiment

**Fig. 6:**
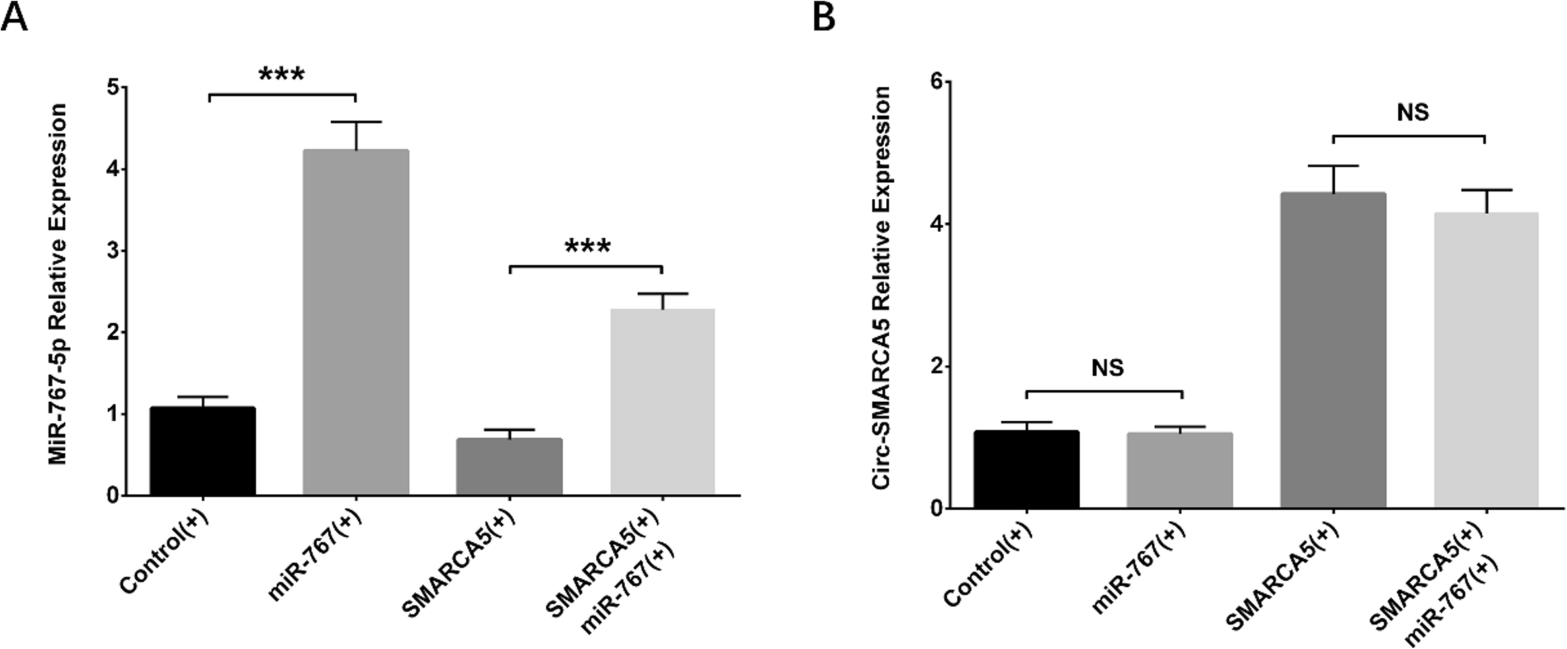
Effect of Circ-SMARCA5 on miR-767-5p expression. MiR-767-5p expression was increased in miR-767 (+) group compared with Control (+) group, as well as in SMARCA5 (+)/miR-767 (+) group compared to SMARCA5 (+) group (**a**). No difference in Circ-SMARCA5 expression was observed between miR-767 (+) group and Control (+) group as well as between SMARCA5 (+) group and SMARCA5 (+)/miR-767 (+) group (**b**). Comparisons of miR-767-5p expression and Circ-SMARCA5 expression were conducted using t test and *P* < 0.05 was considered significant. ***P < 0.001, NS, non-significant. Circ-SMARCA5, Circular RNA SWI/SNF-related matrix-associated actin-dependent regulator of chromatin subfamily A member 5; Control (+), blank overexpression; miR-767 (+), miR-767-5p overexpression; SMARCA5 (+), Circ-SMARCA5 overexpression; SMARCA5 (+)/miR-767 (+), miR-767-5p overexpression & Circ-SMARCA5 overexpression

The interaction between Circ-SMARCA5 and miR-767-5p in RPMI8226 cells was further evaluated by the rescue experiment. We observed that miR-767-5p expression was raised in miR-767 (+) group compared with Control (+) group (*P* < 0.001) and also increased in SMARCA5 (+)/miR-767 (+) group compared with SMARCA5 (+) group (*P* < 0.001) (Fig. 6a). Regarding Circ-SMARCA5, no difference of its expression was observed between miR-767 (+) group and Control (+) group, or between SMARCA5 (+)/miR-767 (+) group and SMARCA5 (+) group (All *P* > 0.05) (Fig. 6b). Therefore, Circ-SMARCA5 adversely regulated miR-767-5p expression while miR-767-5p had no effect on Circ-SMARCA5 expression in RPMI8226 cells.

### Effects of Circ-SMARCA5 and miR-767-5p on cell proliferation and apoptosis in the rescue experiment

**Fig. 7:**
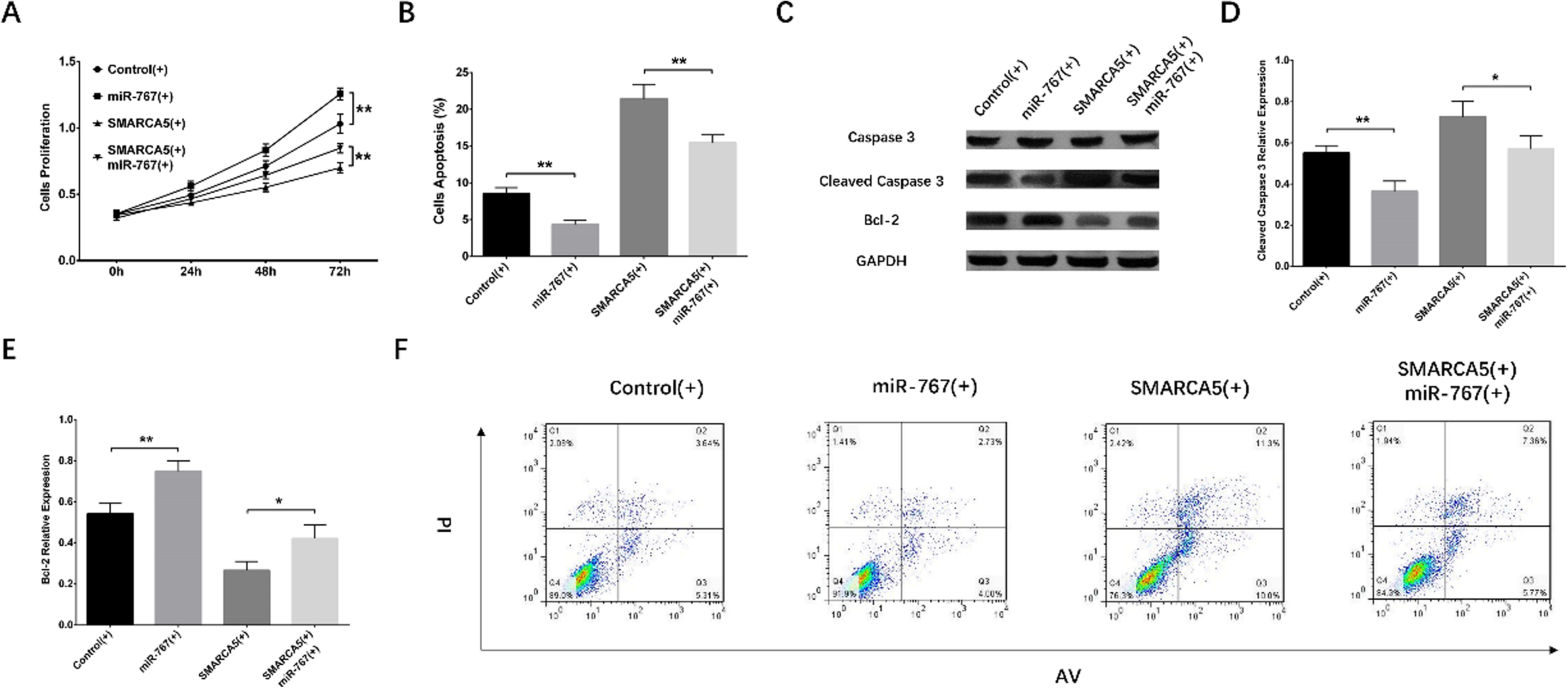
Effect of Circ-SMARCA5 on cell proliferation and apoptosis in the rescue experiment. Cell proliferation was promoted in miR-767 (+) group compared with Control (+) group as well as in SMARCA5 (+)/miR-767 (+) group compared with SMARCA5 (+) group (**a**). Cell apoptosis rate was decreased in miR-767 (+) group compared with Control (+) group as well as in SMARCA5 (+)/miR-767 (+) group compared with SMARCA5 (+) group (**b**, **d**). The expression of cell apoptotic marker Cleaved Caspase 3 was reduced in miR-767 (+) group compared with Control (+) group but raised in SMARCA5 (+)/miR-767 (+) group compared with SMARCA5 (+) group, and for Bcl-2, its expression was promoted in miR-767 (+) group compared with Control (+) group as well as in SMARCA5 (+)/miR-767 (+) group compared with SMARCA5 (+) group (**c**). Comparisons of cell proliferation ability and apoptosis rate were by t test, and *P* < 0.05 was considered significant. **P < 0.01. Round dot represented Control (+) group, square represented miR-767 (+) group, regular triangle represented SMARCA5 (+), inverted triangle represented SMARCA5 (+)/miR-767 (+) group. Circ-SMARCA5, Circular RNA SWI/SNF-related matrix-associated actin-dependent regulator of chromatin subfamily A member 5; miR, micro RNA; Control (+), blank overexpression; miR-767 (+), miR-767-5p overexpression; SMARCA5 (+), Circ-SMARCA5 overexpression; SMARCA5 (+)/miR-767 (+), miR-767-5p overexpression & Circ-SMARCA5 overexpression

At 72 h after transfection, cell proliferation ability was higher in miR-767 (+) group compared with Control (+) group (*P* < 0.01), and higher in SMARCA5 (+)/miR-767 (+) group compared with SMARCA5 (+) group (*P* < 0.01) (Fig. 7a). Cell apoptosis rate was decreased in miR-767 (+) group compared with Control (+) group (*P* < 0.01), as well as in SMARCA5 (+)/miR-767 (+) group compared with SMARCA5 (+) group (*P* < 0.01) (Fig. 7b, f). Western blot assay exhibited that cell apoptotic marker cleaved Caspase 3 expression was reduced in miR-767 (+) group compared with Control (+) group, as well as in SMARCA5 (+)/miR-767 (+) group compared with SMARCA5 (+) group, while Bcl-2 expression was enhanced in miR-767 (+) group compared with Control (+) group, and in SMARCA5 (+)/miR-767 (+) group compared with SMARCA5 (+) group (Fig. 7c, d, e). These results indicated that Circ-SMARCA5 suppressed cell proliferation and promoted cell apoptosis via targeting miR-767-5p in RPMI8226 cells.

### Relative luciferase activity of Circ-SMARCA5

**Fig. 8:**
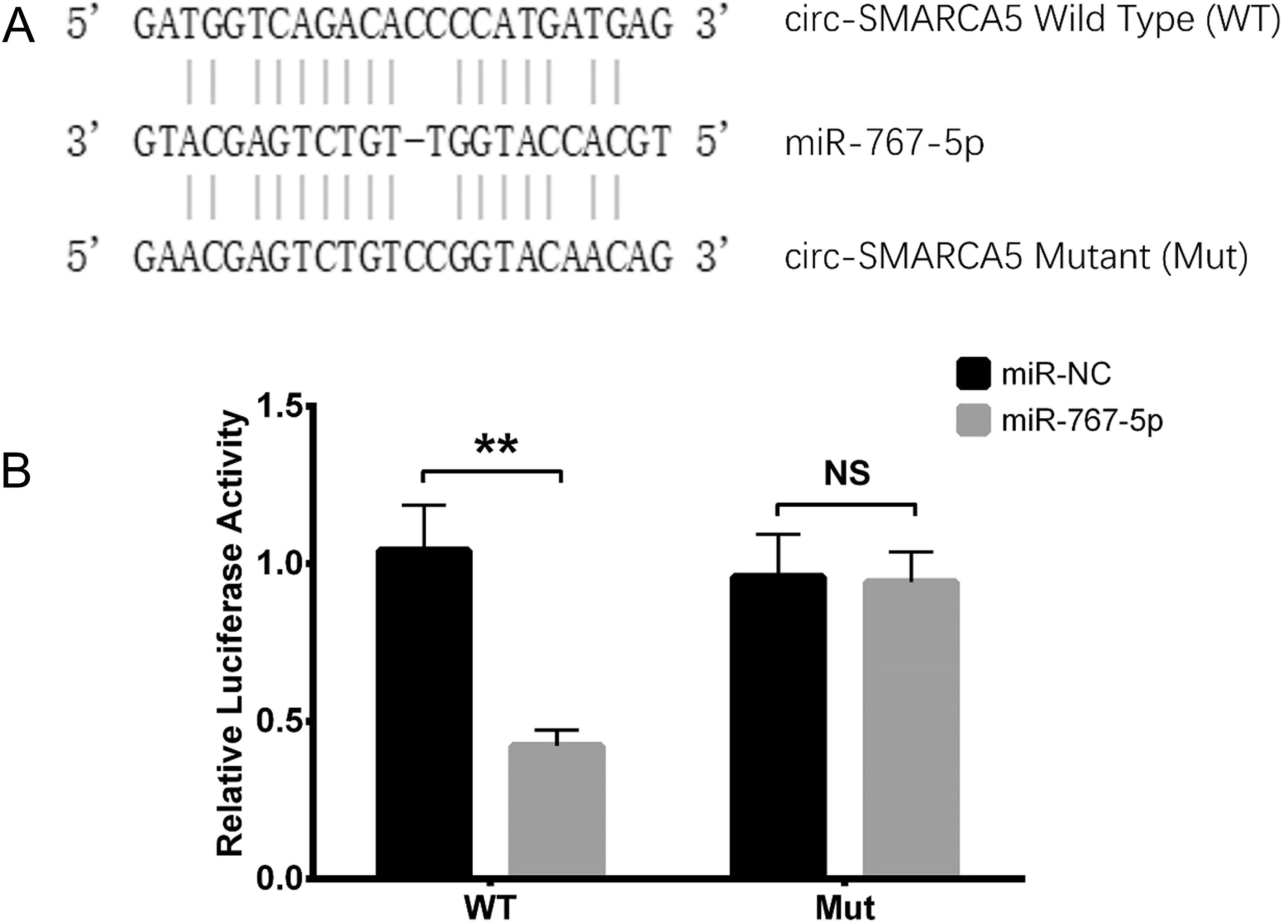
Luciferase reporter assay of Circ-SMARCA5. The sequences of wild type Circ-SMARCA5, mutant type Circ-SMARCA5 and miR-767-5p were shown and the binding sites were lined up (**a**). For wild type Circ-SMARCA5, the relative luciferase activity was decreased in miR-767-5p group compared to miR-NC group, whereas for mutant type Circ-SMARCA5, the relative luciferase activity was similar between miR-767-5p group and miR-NC group (**b**). Comparison of relative luciferase activity between the two groups was conducted using t test, and *P* < 0.05 was considered significant. **P < 0.01, NS, non-significant. Circ-SMARCA5, Circular RNA SWI/SNF-related matrix-associated actin-dependent regulator of chromatin subfamily A member 5; miR, micro RNA

In order to further validate the targeting effect of Circ-SMARCA5 on miR-767-5p, luciferase reporter assay was subsequently conducted to confirm the binding site of Circ-SMARCA5 with miR-767-5p. The sequences of Circ-SMARCA5 wild/mutant type and miR-767-5p were shown and the binding sites were marked in Fig. 8a. For wild type Circ-SMARCA5, the relative luciferase activity was lower in miR-767-5p group compared to miR-NC group (*P* < 0.01), whereas for mutant Circ-SMARCA5, there was no difference in relative luciferase activity between miR-767-5p group and miR-NC group (*P* > 0.05) (Fig. 8b). This validated that Circ- SMARCA5 interacted with miR-767-5p by direct binding in RPMI8226 cells.

## Discussion

Our study discovered that: (1) Circ-SMARCA5 expression was downregulated in MM patients, and its high expression was correlated with lower β2-MG level and ISS stage. (2) Circ-SMARCA5 expression was positively associated with treatment response and survival in MM patients. (3) In vitro experiments disclosed that Circ-SMARCA5 reduced cell proliferation but improved cell apoptosis via sponging miR-767-5p in MM cells.

MM is currently incurable, and its clinical manifestations are not apparent until the oncological events initiate and progress for a decade in most cases, leading to late diagnosis and unfavorable treatment outcomes [2]. Conventional therapy for MM depends on chemotherapy using cytotoxic agents, while the native resistance to drugs is common due to multiple chromosomal abnormities in the pathogenesis of MM, resulting in unfavorable treatment response and survivals in MM patients [1]. Therefore, identifying biomarkers that could predict the risk as well as prognosis of MM will help with timely diagnosis and treatment for MM patients. Moreover, the detailed mechanism underling the development and progression of MM is still not fully understood, thus, studies investigating the mechanism of MM etiopathology are greatly needed.

CircRNAs are widely expressed in human cells with relatively higher expression and more stable structure compared with their linear counterparts, suggesting that CircRNAs could be the ideal biomarkers for human diseases [7]. Accumulating studies have disclosed that CircRNAs might hold missing triggers of tumorigenesis of various cancers including hematopoietic cancers by regulating gene expression [15, 19–21]. For instance, Circ-ANAPC7 is predominantly upregulated in AML by CircRNA microarray analysis and might serve as a promising diagnostic marker for AML [9]. Another study illustrates that Circ_0004277 is notably downregulated in acute myeloid leukemia (AML) and its high expression predicts better treatment response to chemotherapy, acting as one of the diagnostic and treatment targets for AML [10]. In addition, Circ-CBFB has been exhibited to be overly expressed in chronic lymphocytic leukemia (CLL) and serves as a diagnostic and prognostic biomarker for CLL [8]. These previous studies suggest that some CircRNAs play important roles in hematological malignancies and potentially function as promising diagnostic and prognostic markers.

Circ-SMARCA5 is a typical CircRNA derived from exons of SMARCA gene, and very limited studies investigating its role in cancer have been carried out until now [22]. For instance, Circ-SMARCA5 is initially reported to be upregulated in prostate cancer and serves as an oncogene by promoting cancer cell proliferation and inhibiting cell apoptosis [23]. Another research reveals that Circ-SMARCA5 level is reduced in hepatocellular carcinoma (HCC) tissues compared with adjacent tissues and its low expression is correlated with poor clinicopathological features in HCC patients [14]. According to these studies, the role of Circ-SMARCA5 in development of solid tumors is controversial. Besides, it is still unknown whether Circ-SMARCA5 is involved in MM or not. In vitro studies have shown that SMARCA5 gene is dysregulated in hematopoietic progenitors that could also potentially develop into MM cells, therefore, we speculated that Circ-SMARCA5, which is encoded from SMARCA5 gene, might play critical roles in MM etiology [15, 24]. In this study, we compared the expression of Circ-SMARCA5 in MM patients with that of controls, which revealed that Circ-SMARCA5 was downregulated in MM patients compared to controls. Moreover, we also found that Circ-SMARCA5 high expression was correlated with lower β2-MG level and ISS stage in MM patients. Here are several possible explanations: (1) Circ-SMARCA5 might be one of the anti-oncogenes whose expression was inhibited by the onset of MM, therefore, Circ-SMARCA5 expression was downregulated in MM patients. (2) Circ-SMARCA5 might participate in cell cycle regulation by inhibiting cell proliferation and promoting cell apoptosis in MM cells to attenuate disease development and progression, thereby reduced disease severity in MM patients, which was validated in our followed in vitro experiments.

Additionally, we also investigated the prognostic value of Circ-SMARCA5 in MM patients and discovered that Circ-SMARCA5 high expression was correlated with better treatment response to chemotherapy and longer survival, indicating that patients with Circ-SMARCA5 high expression were more sensitive to clinical treatment. These could be due to that: (1) Circ-SMARCA5 might function as an anti-tumor gene whose high expression was associated with lower disease severity. Thus, MM patients with higher Circ-SMARCA5 presented with less advanced disease (lower ISS stage or Durie-Salmon stage), thereby leading to better treatment outcomes, which was further supported by results from Multivariate Cox’s regression analysis in our study. (2) Circ-SMARCA5 might affect cells sensitivity to cytotoxic drugs, thereby resulting in better treatment response to chemotherapy and prolonging survival in MM patients. However, further validation is needed focusing on the influence of Circ-SMARCA5 on chemoresistance. Moreover, the sample size of this study was relatively small, which might influence statistical power. Also, the follow-up time for evaluating survivals was relatively short, therefore the effect of Circ-SMARCA5 on long-term prognosis in MM patients was still not clear in this study.

The mechanisms of CircRNAs in oncogenesis of human malignancies are still not well recognized, nonetheless, studies have discovered that CircRNAs act as miRNA sponges to negatively regulate miRNAs and protein transcription, which assist with understanding of CircRNAs in human cancers [10, 25]. For instance, Circ_0000673 inhibits cell proliferation but promotes cell apoptosis of gastric cancer cells via adversely regulating miR-532-5p [26]. As for breast cancer (BC), Circ_0008039 functions as competing endogenous RNA and increases cell proliferation as well as cells migration via sponging miR-432-5p and altering E2F3 [27]. In hematological malignancies, Circ-CBFB is overexpressed in CLL and is a sponge of miR-607, which promotes CLL cell proliferation but suppresses cell apoptosis via activating FZD3 and the following Wnt/β-catenin pathway [8]. Also, a bioinformatic analysis elucidates that Circ-ANAPC7, which is increasingly expressed in AML, facilitates AML pathogenesis by serving as a sponge of miR-181 family [9]. Although previous functional studies have illustrated the mechanisms of several CircRNAs in hematological malignancies, the mechanism of Circ-SMARCA5, a typical CricRNA, is still not clear in these cancers, nor in MM. In order to further understand how Circ-SMARCA5 acted in MM, we performed in vitro experiments and disclosed that Circ-SMARCA5 was downregulated in MM cells lines, and it inhibited proliferation but promoted apoptosis of MM cells. This suggested that Circ-SMARCA5 might work as an anti-oncogene and be a potential therapeutic target for MM.

MiRNAs are single-stranded non-coding RNAs with length of 19–22 nucleotides, which are common targets of CircRNAs and are involved in cells growth, proliferation, apoptosis and differentiation by incomplete base pairing with messenger RNAs (mRNAs) [28]. MiR-767-5p is a member of miR-767 that are located on chromosome X (location: NC_000023.11 (152,393,421..152393529, complement)) [22]. In human melanoma, miR-767 is shown to promote cell proliferation by suppressing cylindromatosis [29]. Another functional experiment displays that aberrant activation of miR-767 devotes to tumor epigenesis in lung cancer via repressing TET1/3 mRNA and regulating genomic 5-methylcytosines to 5-hydroxymethylcytosines levels [11]. These previous studies disclose the oncogenic role of miR-767 in several human cancers, whereas in MM, the function of miR-767 still lacks investigation, and only two studies have been disclosed: the first study observed that miR-767-5p was upregulated in both tissue (two folds) and plasma samples (four folds) from MM patients compared to normal control, and it promotes MM cells (H929 and MM.1S) progression via regulating MAPK4 pathway [30]; the second study observed that miR-767-5p was overexpression in extramedullary relapse MM samples compared to non-extramedullary relapse MM samples [31]. In order to explore the potential target miRNA of Circ-SMARCA5 in MM, we referred to Circular RNA Interactome database (https://Circinteractome.nia.nih.gov/) and miRanda database (http://www.microrna.org/microrna/home.do) and found that miR-767-5p might be a potential target for Circ-SMARCA5 in etiology of MM. To testify this hypothesis, we further performed rescue experiments, which illuminated that Circ-SMARCA5 suppressed cell proliferation but facilitated cell apoptosis by adversely regulating miR-767-5p in RPMI8226 cells. Moreover, the direct binding between Circ-SMARCA5 and miR-767-5p was illustrated by luciferase reporter assay.

## Conclusions

In conclusion, Circ-SMARCA5 is downregulated and correlated with lower β2-MG level and ISS stage as well as better prognosis in MM patients, and it inhibits proliferation but promotes apoptosis of MM cells via directly sponging miR-767-5p.

## Additional files

Additional file 1: **Table S1.** Primers applied in qPCR. (DOCX 14 kb)

Additional file 2: **Table S2.** Antibodies applied in Western Blot. (DOCX 14 kb)

Additional file 3: **Figure S1**. Validation for the regulatory effect of Circ-SMARCA5 on miR-767-5p. Description of data: The expression of miR-767-5p was decreased in SMARCA5 (+) group compared with Control(+) group and increased in SMARCA5(−) group compared with Control(−) group in NCI-H299 cells (A), U226 cells (B), OPM2 cells (C) and JJN3 cells (D). Comparisons of miR-767-5p expressions were performed using t test and *P* < 0.05 was considered significant. *P < 0.05, <**P < 0.01, ***P < 0.001. Control (+), blank overexpression; SMARCA5 (+), Circ-SMARCA5 overexpression; Control (−), blank shRNA; SMARCA5 (−), Circ-SMARCA5 shRNA; miR, micro RNA. (DOCX 320 kb)

## Data Availability

The datasets used and/or analysed during the current study are available from the corresponding author on reasonable request.

## Abbreviations

ALB: Albumin
CI: Confidence interval
Circ-SMARCA5: Circular RNA SWI/SNF-related matrix-associated actin-dependent regulator of chromatin subfamily A member 5
CR: Complete remission
Hb: Hemoglobin
HCC: Hepatocellular carcinoma
Ig: Immunoglobulin
ISS: International Staging System
LDH: Lactate dehydrogenase
miR: micro RNA
MM: Multiple myeloma
NS: Non-significant
OR: Odds ratio
ORR: Overall response rate
qPCR: quantitative polymerase chain reaction
ROC: Receiver operating characteristic
Scr: Serum creatinine
β2-MG: Beta-2-microglobulin
