# Supplementary material for: Circ-SMARCA5 suppresses progression of multiple myeloma by targeting miR-767-5p
Source: BMC Cancer. 2019 Oct 10;19:937. doi: 10.1186/s12885-019-6088-0 (PMC6785934; doi:10.1186/s12885-019-6088-0)
Supplement: Supplementary file 1 — Table S1. Primers applied in qPCR. (DOCX 14 kb) [file 12885_2019_6088_MOESM1_ESM.docx]

**Additional file 1: Table S1** Primers applied in qPCR

| Gene | Forward Primer (5’-3’) | Reverse Primer (5’-3’) |
| --- | --- | --- |
| Circ-SMARCA5 | ATACTCAACTCAGCAGGCAAGAT | TTACTACATCAGCAGTCGCAAGA |
| MiR-561 | ACACTCCAGCTGGGATCAAGGATCTTAAACTT | TGTCGTGGAGTCGGCAATTC |
| MiR-616 | ACACTCCAGCTGGGCATGCTACTCAAAACCCTTCAGTG | TGTCGTGGAGTCGGCAATTC |
| MiR-767-5P | CTCAACTGGTGTCGTGGAGTCGGCAATTCAGTTGAGCATGCT | TGTCGTGGAGTCGGCAATTC |
| GAPDH | GAGTCCACTGGCGTCTTCAC | ATCTTGAGGCTGTTGTCATACTTCT |
| U6 | CTCGCTTCGGCAGCACATATACTA | ACGAATTTGCGTGTCATCCTTGC |

File name: Supplementary Table 1.

Title of data: Primers applied in qPCR.

Description of data: The Forward Primer (5’-3’) and Reverse Primer (5’-3’) of Circ-SMARCA5, miR-561, miR-616, miR-767-5P, GAPDH and U6 in qPCR analysis.
