# Supplementary material for: Circ-SMARCA5 suppresses progression of multiple myeloma by targeting miR-767-5p
Source: BMC Cancer. 2019 Oct 10;19:937. doi: 10.1186/s12885-019-6088-0 (PMC6785934; doi:10.1186/s12885-019-6088-0)
Supplement: Supplementary file 2 — Table S2. Antibodies applied in Western Blot. (DOCX 14 kb) [file 12885_2019_6088_MOESM2_ESM.docx]

**Additional file 2: Table S2** Antibodies applied in Western Blot

| Antibody | Company | Dilution |
| --- | --- | --- |
| **Primary Antibody** |  |  |
| Rabbit polyclonal to Caspase 3 | CST (USA) | 1:1000 |
| Rabbit polyclonal to Cleaved Caspase 3 | CST (USA) | 1:1000 |
| Rabbit polyclonal to Bcl 2 | CST (USA) | 1:1000 |
| Rabbit monoclonal to GAPDH | CST (USA) | 1:1000 |
| **Secondary Antibody** |  |  |
| Goat Anti-Rabbit IgG H&L (HRP) | Abcam (HK) | 1:2000 |

File name: Supplementary Table 2.

Title of data: Antibodies applied in Western Blot.

Description of data: The primary and secondary antibodies applied in Western blot analysis.
