# Supplementary material for: Circ-SMARCA5 suppresses progression of multiple myeloma by targeting miR-767-5p
Source: BMC Cancer. 2019 Oct 10;19:937. doi: 10.1186/s12885-019-6088-0 (PMC6785934; doi:10.1186/s12885-019-6088-0)
Supplement: Supplementary file 3 — Figure S1. Validation for the regulatory effect of Circ-SMARCA5 on miR-767-5p. Description of data: The expression of miR-767-5p was decreased in SMARCA5 (+) group compared with Control(+) group and increased in SMARCA5(−) group compared with Control(−) group in NCI-H299 cells (A), U226 cells (B), OPM2 cells (C) and JJN3 cells (D). Comparisons of miR-767-5p expressions were performed using t test and P < 0.05 was considered significant. *P < 0.05, <**P < 0.01, ***P < 0.001. Control (+), blank overexpression; SMARCA5 (+), Circ-SMARCA5 overexpression; Control (−), blank shRNA; SMARCA5 (−), Circ-SMARCA5 shRNA; miR, micro RNA. (DOCX 320 kb) [file 12885_2019_6088_MOESM3_ESM.docx]

**
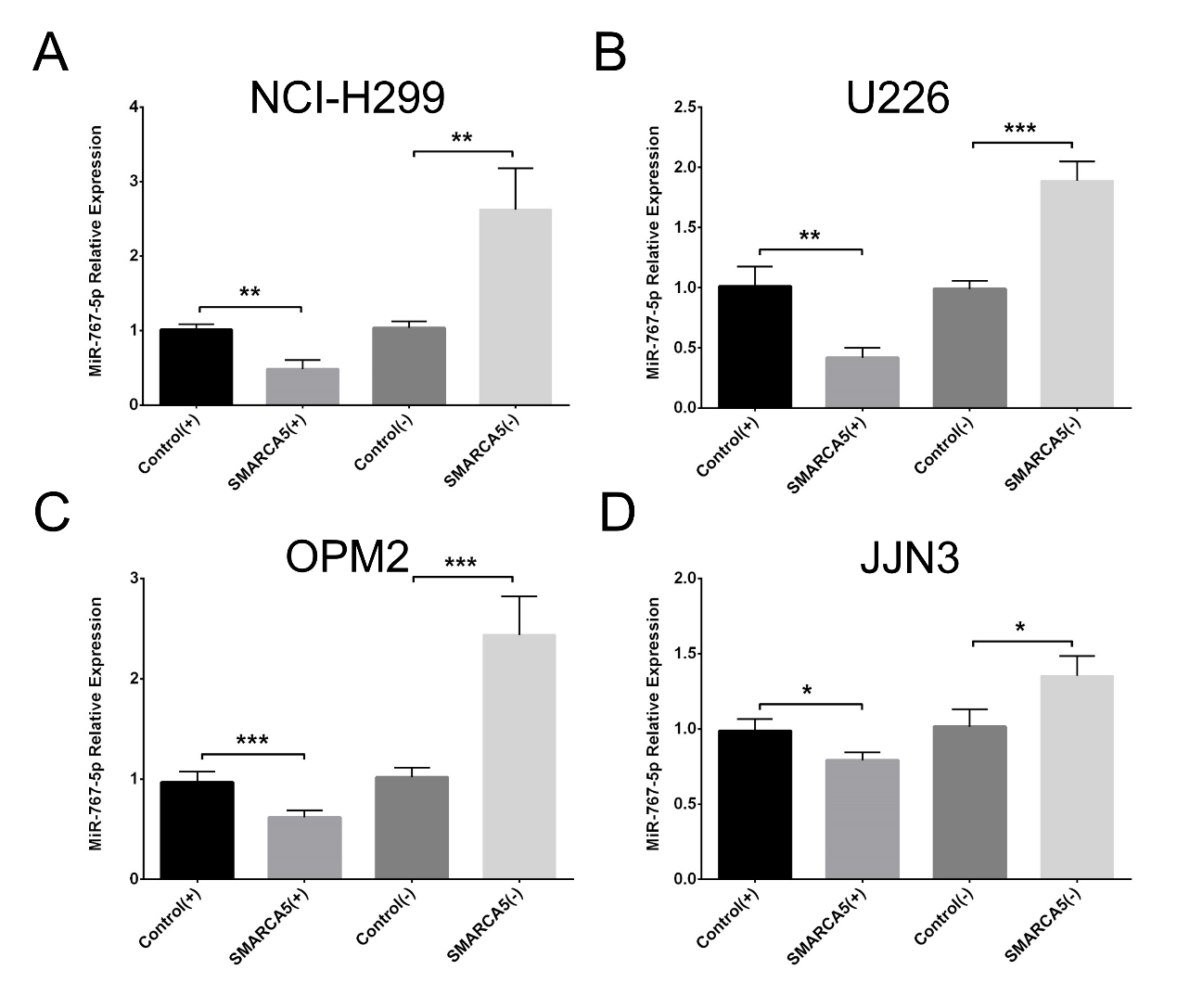
Additional file: 3** Figure S1**.** Title of data: Validation for the regulatory effect of Circ-SMARCA5 on miR-767-5p. Description of data: The expression of miR-767-5p was decreased in SMARCA5 (+) group compared with Control(+) group and increased in SMARCA5(-) group compared with Control(-) group in NCI-H299 cells (A), U226 cells (B), OPM2 cells (C) and JJN3 cells (D). Comparisons of miR-767-5p expressions were performed using t test and P<0.05 was considered significant. *P<0.05, <**P<0.01, ***P<0.001. Control (+), blank overexpression; SMARCA5 (+), Circ-SMARCA5 overexpression; Control (-), blank shRNA; SMARCA5 (-), Circ-SMARCA5 shRNA; miR, micro RNA.
